# Genetic Polymorphisms of *CYP2E1*, *GSTP1*, *NQO1 *and *MPO *and the Risk of Nasopharyngeal Carcinoma in a Han Chinese Population of Southern China

**DOI:** 10.1186/1756-0500-3-212

**Published:** 2010-07-27

**Authors:** Xiuchan Guo, Yi Zeng, Hong Deng, Jian Liao, Yuming Zheng, Ji Li, Bailey Kessing, Stephen J O'Brien

**Affiliations:** 1 State Key Laboratory for Infectious Diseases Prevention and Control, Institute for Viral Disease Control and Prevention, Chinese Centers for Disease Control and Prevention, Beijing, China; 2 Laboratory of Genomic Diversity, SAIC-Frederick, Inc., National Cancer Institute, Frederick, MD 21702, USA; 3 Wuzhou Red Cross Hospital, Wuzhou 543002, Guangxi, China; 4 Cangwu Institute for Nasopharyngeal Carcinoma Control and Prevention, Wuzhou, Guangxi, China; 5 Laboratory of Genomic Diversity, National Cancer Institute, Frederick, MD 21702, USA

## Abstract

**Background:**

Southern China is a major area for endemic nasopharyngeal carcinoma (NPC). Genetic factors as well as environmental factors play a role in development of NPC. To investigate the roles of previously described carcinogen metabolism gene variants for NPC susceptibility in a Han Chinese population, we conducted a case-control study in two independent study population groups afflicted with NPC in Guangdong and Guangxi Provinces of southern China.

**Methods:**

Five single nucleotide polymorphisms (SNPs) of *CYP2E1*-rs2031920, *CYP2E1*-rs6413432, *GSTP1*-rs947894, *MPO*-rs2333227 and *NQO1*-rs1800566 were genotyped by PCR-based RFLP, sequencing and TaqMan assay in 358 NPC cases and 629 controls (phase I cohort). Logistic regression analysis was used to estimate odds ratios (OR) and 95% confidence intervals (CI). To confirm our results, sixteen tag SNPs for *GSTP1*, *MPO*, *NQO1 *(which 100% covered these genes), and 4 functional SNPs of *CYP2E1 *were genotyped in another cohort of 213 NPC cases and 230 controls (phase II cohort).

**Results:**

No significant associations in NPC risk were observed for the five polymorphisms tested in the phase I cohort. In an additional stratified analysis for phase I, there was no significant association between cases and controls in NPC high risk population (EBV/IgA/VCA positive population). Analysis of 14 tagging SNPs within the same genes in an independent phase II cohort were in agreement with no SNPs significantly associated with NPC.

**Conclusions:**

Our results suggest that polymorphism of *CYP2E1*, *GSTP1*, *MPO *and *NQO1 *genes does not contribute to overall NPC risk in a Han Chinese in southern China.

## Background

Nasopharyngeal carcinoma (NPC) is rare in most regions of the world; however it is a common cancer in southern China, especially in Guangdong and Guangxi Provinces. The incidence rate of NPC for males is more than 20 per 100,000 and up to 25-40 per 100,000 in some areas bordering the Xijiang River and Pearl River drainages in these two provinces[1,2]. Both environmental and genetic factors have been associated with the risk of developing NPC. Epidemiologic studies have indicated a strong influence for Epstein-Barr virus (EBV) infection, consumption of salt-preserved fish, exposure to domestic wood cooking fires, and exposure to occupational solvents in the risk for NPC[3,4]. Accumulating data have shown that genetic polymorphisms of the genes responsible for the xenobiotic enzymes which metabolize carcinogenic compounds underlie individual variations in cancer risk[5]. Most carcinogens undergo activation by Phase I enzymes, often as an oxidation reaction, and detoxification by Phase II enzymes.

*CYP2E1 *is a Phase I enzyme included in the cytochrome P450 superfamily. *CYP2E1 *plays a major role in the metabolic activation of nitrosamines. The RsaI polymorphism of *CYP2E1 *(rs2031920) in the promoter region has been reported to be associated with increased risk of lung cancer and lymphomas[6,7]. Two studies have associated this polymorphism with NPC development[8,9]. Glutathione S-transferases (GSTs) are a group of enzymes involved in Phase II detoxification of carcinogens. *GSTP1 *is a major GST isoform. The Ile to Val substitution at codon 105 of exon 5 variant in *GSTP1 *(rs947894) has been investigated for associations with breast and lung cancer in several studies[10,11]. Four of 22 studies showed a significantly higher risk for head and neck cancers in persons with the Ile/Val and/or Val/Val genotypes than in those with the Ile/Ile genotypes[5].

*NQO1 *is an enzyme previously shown to detoxify a number of natural and synthetic compounds and, conversely, to activate certain anticancer agents[12]. A single nucleotide polymorphism (SNP) C to T at position 609 (rs1800566) in the *NQO1 *coding region (replacing proline with serine at codon 187) leads to lower enzyme activity and has been shown to be associated with increased risk of myeloid leukemia and bladder carcinoma[12,13]. Myeloperoxidase (*MPO*) is an endogenous oxidant enzyme that generates reactive oxygen species (ROS). A single G-463A base transition in the *MPO *promoter region was identified at the SP1 binding site, which might modify carcinogen metabolism. The 463A allele has been associated with reduced mRNA expression and its transcription activity is approximately 25 times lower than the 463G allele *in vitro *due to reduced binding of SP1[14,15]. This polymorphism has also been reported to be associated with risk for several human cancers including liver, ovarian, bladder and lung cancer [16-23].

We conducted a case-control study with 358 NPC cases and 629 controls from southern China to determine if these previously implicated polymorphisms were associated with NPC development. We also investigated sixteen adjacent tagging SNPs of *GSTP1*, *MPO *and *NQO1 *(which covered 100% haplotype variation within these genes), and four SNPs of *CYP2E1 *(one of which covered *CYP2E1*-RsaI polymorphisms) in another independent case-control cohort with 213 NPC cases and 230 controls.

## Methods

### Patients and controls

The NPC cohorts were recruited from areas along the Xijiang River in Guangdong and Guangxi provinces of southern China in two collection phases. The phase I participants were recruited from April 2000 to June 2001. NPC cases were incident or prevalent biopsy-confirmed NPC cases with or without IgA antibodies to EBV capsid antigens (EBV/IgA/VCA). The controls were the case's spouse or geographically matched residents who were EBV/IgA/VCA positive (IgA+) or EBV/IgA/VCA negative (IgA-) and NPC free at the time of study enrollment. The phase II participants were recruited from November 2004 to October 2005. The cases were incident or prevalent biopsy-confirmed NPC cases who were IgA+. The controls were IgA+, NPC free at the time of study enrollment and matched to NPC cases on age, sex, and district/township of residence. NPC cases were hospitalized patients at Wuzhou Red Cross Hospital in Wuzhou City and outpatients at Cangwu Institute for NPC Control and Prevention in Cangwu County. All participants self-identified as Han Chinese and self-reported 6 or more months of residency in Guangdong or Guangxi Province of China. EBV/IgA/VCA and EBV/IgA/EA were confirmed by serological testing at the time of study enrollment. Blood samples were obtained from 358 NPC cases (239 males and 119 females) and 629 controls (271 males and 358 females) in the phase I cohort; the mean age was 45 years (± 11 years standard deviation [SD]) for cases and 46 years (± 10 years SD) for controls. Blood samples were collected from 213 IgA+ NPC cases (155 males and 58 female) and 230 IgA+ controls (179 males and 51 females) in the phase II cohort; the mean age was 45 years (± 9 years SD) for cases and 45 years (± 10 years SD) for controls (see Table 1). We excluded persons of minority ethnicity and those who had blood relatives in either the cases or controls. We also did not allow overlap in participation between phase I and phase II; that is, persons could not be in both cohorts. Institutional review board approval was obtained from all participating institutions and informed consent was obtained from each study participant.

**Table 1:** Characteristics of participants in a study of nasopharyngeal carcinoma (NPC) in southern China

	Phase I		Phase II	
		
	Cases	Controls	Cases	Controls
Age (years)	45 ± 11.4 (SD)	46 ± 9.7 (SD)	45 ± 9.1 (SD)	45 ± 9.7 (SD)
Male, %	66.8 (239/358)	43.1 (271/629)	72.8 (155/213)	77.8 (179/230)
IgA+*, %	95.3 (341/358)	44.5 (280/629)	100 (213/213)	100 (230/230)

Total	358	629	213	230

Age was the age at diagnosis of NPC for cases and age of enrollment for controls.

SD = standard deviation.

* Positive for IgA antibodies to Epstein-Barr virus capsid antigen.

### Genomic DNA extraction

Phase I DNA was extracted from whole blood or lymphoblastoid cell lines (LCL) using QIAamp DNA blood maxi kit (Qiagen, Valencia, CA, catalog # 51194). More than 80% of the genotypes were determined from DNA directly extracted from whole blood. Phase II DNA was extracted from whole blood by traditional phenol/Chloroform method with Phase Lock Gel tube (Qiagen, MaXtract High Density, catalog # 129065).

### SNP selection and genotyping

For the phase I cohort, five functional SNPs, which previously were reported associated with NPC or other cancers, were interrogated for four xenobiotic metabolic enzyme genes-- *CYP2E1*-RsaI (rs2031920), *CYP2E1*-DraI (rs6413432), *GSTP1 *Ile105Val (rs947894), *MPO *G-463A (rs2333227) and *NQO1 *C609T (rs1800566). For phase I samples, *CYP2E1*-rs6413432, *GSTP1*-rs947894 and *MPO*-rs2333227 were genotyped by using a commercially available TaqMan SNP genotyping assay and GeneAmp PCR System 9700 (Applied Biosystems, Foster City, CA, USA), in accordance with the manufacturer's instructions. Sequence detection software was used for allelic discrimination. *CYP2E1*-rs2031920 was genotyped by sequence according to the method available in the SNP500Cancer Database[24]. *NQO1*-rs1800566 was genotyped using PCR-restriction fragment length polymorphism (RFLP) assay; the polymerase chain reactions (PCR) were performed in 15 μl volumes containing 10 mM Tris-HCL buffer, 50 mM KCL, 2.0 mM MgCl2, 0.2 mM dNTP, 0.25 mM of each of the primers (forward primer: GAGACGCTAGCTCTGAACTGAT, reverse prime: ATTTGAATTCGGGCGTCTGCTG), 40ng genomic DNA and 5 units of Taq polymerase with thermal cycling conditions of 95°C for 8 minutes followed by 35 cycles at 94°C for 30 seconds, 62°C for 30 seconds, 72°C for 45 seconds, and then a final extension of 72°C for 8 minutes. The PCR product was digested by Hinf1 enzyme. The variants (size of bands for each allele) were determined by a 4% agarose gel assay in 0.5× TBE.

For the phase II cohort, sixteen tagging SNPs for the same metabolism enzyme genes were selected, which covered 5,000 base pairs (bp) upstream and 1,000 bp downstream of these genes. Because two tagging SNPs in the *CYP2E1 *gene had a low design score on Illumina measurements, we selected four other SNPs located in the locus or coding region. One of them, rs3813867, tracked *CYP2E1*-RsaI (rs2031920) that was previously reported associated with NPC[8]. For phase II samples, sixteen SNPs including rs7927381, rs6591256 and rs947895 for *GSTP1*; rs2071409 and rs2243828 for *MPO*; rs10517, rs1800566, rs4986998, rs689452, rs2917667, rs2917666 and rs1469908 for *NQO1*; and rs3813867, rs2070673, rs41299426 and rs41299434 for *CYP2E1 *were genotyped by Illumina GoldenGate assay. Fourteen SNPs (all except rs41299426 and rs41299434 of *CYP2E1 *which were excluded due to low minimum allele frequency) were analyzed as described below.

### Statistical analysis

Hardy-Weinberg equilibrium (HWE) compliance was independently tested for each polymorphism. For phase I, differences between predicted genotype frequencies based upon binomially distributed allele frequency and observed genotype frequencies were tested using a χ^2 ^goodness of fit test of co-dominant model. Odd ratios (OR) and 95% confidence intervals (CIs) were computed by logistic regression. In order to investigate the influence of EBV/IgA/VCA antibody status, we also analyzed the associations between polymorphisms and the occurrence of NPC in an EBV/IgA/VCA positive subpopulation. The results of the phase I analysis were adjusted for age and gender. For the phase II cohort, the linear trend chi-square tests were used to test for additive allele effects on the disease penetrance. Odds ratios are calculated based on the 2 × 2 table of allele-by-trait counts. All statistics were calculated in the statistical package SAS/SAS Genetics version 9.1.3.

## Results

### Association results in phase I cohort

Five SNPs in four metabolism enzyme loci genes were genotyped in 358 nasopharyngeal carcinoma cases and 629 controls. Over 95% of NPC cases (n = 341) and 45% of the controls were positive for EBV/IgA/VCA antibodies in the phase I cohort (Table 1). Each of the five SNPs demonstrated conformance to Hardy-Weinberg equilibrium in the phase I cohort. The rates of successful genotyping were 81.1% for *GSTP1*-rs947894, 88.4% for *MPO*-rs2333227, 95.2% for *CYP2E1*-rs6413432, 98.0% for *NQO1*-rs1800566 and 99.3% for *CYP2E1*-rs2031920. The genotype distributions, odds ratios, 95% confidence intervals and p-values for the association of five polymorphisms in cases and controls are shown in Table 2. No significant differences in the frequencies of genotypes for the five SNPs were observed between cases and controls. We also stratified the analysis by EBV/IgA/VCA status to test for joint effects. No significant differences in frequencies of genotypes for these SNPs were found between EBV/IgA/VCA-positive cases and controls in our study population (Table 2).

**Table 2:** Odds ratios for the association of five polymorphisms with risk of nasopharyngeal carcinoma (NPC) in southern China (Phase I cohort)

Gene-SNP	Genotype	Cases (%)	Controls (%)	OR	95%CI	*p *value
All subjects*		(n = 358)	(n = 629)			
CYP2E1-rs2031920	CC	228 (64.0)	412 (66.0)	1.0		
	CT	108 (30.3)	186 (29.8)	1.06	0.78-1.42	0.72
	TT	20 (5.6)	26 (4.2)	1.21	0.63-2.34	0.57
CYP2E1-rs6413432	TT	188 (56.0)	360 (59.6)	1.0		
	AT	121 (36.0)	202 (33.4)	1.10	0.82-1.49	0.52
	AA	27 (8.0)	42 (7.0)	1.07	0.61-1.85	0.82
GSTP1-rs947894	AA	181 (65.1)	346 (66.3)	1.0		
	AG	84 (30.2)	159 (30.5)	0.98	0.70-1.37	0.91
	GG	13 (4.7)	17 (3.3)	1.41	0.65-3.03	0.39
MPO-rs2333227	GG	223 (69.0)	381 (69.3)	1.0		
	GA	93 (28.8)	157 (28.6)	0.95	0.69-1.30	0.74
	AA	7 (2.2)	12 (2.2)	1.15	0.43-3.07	0.78
NQO1-rs1800566	CC	96 (27.7)	169 (27.3)	1.0		
	CT	159 (45.8)	320 (51.6)	0.87	0.63-1.21	0.41
	TT	92 (26.5)	131 (21.1)	1.18	0.81-1.72	0.39
EBV/IgA/VCA+**		(n = 341)	(n = 280)			
CYP2E1-rs2031920	CC	214 (63.1)	186 (66.9)	1.0		
	CT	105 (31.0)	82 (29.5)	1.10	0.77-1.58	0.59
	TT	20 (5.9)	10 (3.6)	1.39	0.61-3.15	0.43
CYP2E1-rs6413432	TT	175 (54.7)	160 (58.8)	1.0		
	AT	118 (36.9)	93 (34.2)	1.12	0.79-1.60	0.52
	AA	27 (8.4)	19 (7.0)	1.03	0.54-1.98	0.92
GSTP1-rs947894	AA	172 (65.4)	168 (69.7)	1.0		
	AG	79 (30.0)	67 (27.8)	1.09	0.73-1.63	0.66
	GG	12 (4.6)	6 (2.5)	1.80	0.65-5.01	0.26
MPO-rs2333227	GG	217 (70.2)	159 (66.5)	1.0		
	GA	85 (27.5)	72 (30.1)	0.84	0.57-1.23	0.37
	AA	7 (2.3)	8 (3.4)	0.77	0.27-2.22	0.63
NQO1-rs1800566	CC	91 (27.6)	73 (26.6)	1.0		
	CT	151 (45.8)	139 (50.6)	0.88	0.60-1.31	0.53
	TT	88 (26.7)	63 (22.9)	1.11	0.70-1.74	0.66

OR = odds ratio.

CI = confidence interval.

*Cases and controls in this group were a mix of IgA+ and IgA- individuals.

**Cases and controls in this group were only IgA+ individuals.

### Association results in phase II cohort

All cases (N = 213) and controls (n = 230) in the phase II cohort were positive for EBV/IgA/VCA antibodies (Table 1). Genotypes were obtained for more than 95% of the participants for 16 SNPs in this cohort. The rs41299426 and rs41299434 of *CYP2E1 *with minor allele frequencies less than 1% were excluded from analysis. The genotype frequencies of the other 14 SNPs conformed to Hardy-Weinberg equilibrium. Table 3 lists the information of SNPs, minor allele frequencies (MAF), the association of ORs, 95% CIs and p values between cases and controls. There were no significant differences for these polymorphisms between cases and controls in the NPC phase II cohort (*p *= 0.08-0.89).

**Table 3:** Association of *CYP2E1*, *GSTP1*, *MPO *and *NQO1 *with risk of nasopharyngeal carcinoma (NPC) in an IgA+ population in southern China (Phase II cohort)

SNP-ID	Allele	SNP-Type	MAF†	Cases	Controls	OR	95%CI	Trend p
CYP2E1								
rs3813867	C/G	Locus-region	0.219	207	226	1.34	0.97-1.86	0.08
rs2070673	A/T	Locus-region	0.450	212	229	0.95	0.73-1.24	0.69
GSTP1								
rs7927381	C/T	~4k upstream	0.163	211	230	1.15	0.80-1.64	0.46
rs6591256	A/G	Locus-region	0.175	210	226	1.24	0.87-1.76	0.24
rs947895	A/C	Locus-region	0.182	208	226	0.98	0.69-1.38	0.89
MPO								
rs2071409	A/C	intron	0.183	207	229	0.83	0.59-1.17	0.28
rs2243828	A/G	downstream	0.175	200	221	0.74	0.52-1.06	0.12
NQO1								
rs10517	C/T	mRNA-UTR	0.296	210	224	0.83	0.62-1.11	0.21
rs1800566	C/T	Conding-nonsyn	0.474	205	225	0.90	0.69-1.18	0.46
rs4986998	C/T	intron	0.041	205	227	1.05	0.53-2.06	0.89
rs689452	C/G	intron	0.360	213	230	0.97	0.74-1.28	0.83
rs2917667	C/T	downstream	0.360	213	230	1.03	0.78-1.36	0.81
rs2917666	C/G	downstream	0.156	213	230	0.88	0.61-1.27	0.50
rs1469908	A/G	downstream	0.164	209	230	1.16	0.81-1.66	0.41

OR = odds ratio.

CI = confidence interval.

^†^Minor allele frequency

## Discussion

Five genetic variants of *CYP2E1*, *GSTP1*, *MPO *and *NQO1 *were genotyped in 358 NPC cases and 629 controls (phase I cohort) from NPC high incidence regions in southern China. No significant association with risk of NPC was observed (Table 2). A previous study found the highest risk population for NPC in southern China is EBV/IgA/VCA antibody positive (IgA+)[4]. To investigate potential influence of factors exclusive of EBV/IgA/VCA antibody development, we stratified the analysis in the phase I cohort to compare 341 NPC cases with IgA+ to 280 IgA + controls. Still, no significant association of genotypes examined with NPC risk was noted (Table 2). To attempt replication from a previous report of a 2.6-fold increased risk of NPC among individuals homozygous for TT of *CYP2E1*-RsaI (rs2031920) variant[8], the tagging SNPs for *GSTP1*, *MPO*, and *NQO1 *(which 100% covered these genes) and four SNPs of *CYP2E1 *(which covered *CYP2E1*-RsaI (rs2031920) variant) were genotyped in a second independent cohort of 213 IgA+ NPC cases and 230 IgA+ controls (phase II cohort). Similarly no significant association was found between polymorphisms tracking common SNP variation across the four genes and risk of NPC (table 3).

Our results did show a trend for slightly increased risk of NPC with T allele of *CYP2E1*-RsaI (OR = 1.06 for CT and OR = 1.21 for TT), and a slightly greater risk in individuals with IgA antibodies to EBV capsid antigen (OR = 1.10 for CT and OR = 1.39 for TT), but no statistically significant association in either situation (Table 2). The replication result of *CYP2E1*-rs3813867 which covered *CYP2E1*-RsaI from the phase II cohort indicated a similar trend (OR = 1.34); again this did not reach the level of statistical significance (Table 3). Although numerous studies have examined the association of the polymorphisms for *CYP2E1*, *GSTP1*, *MPO *and *NQO1 *genes with various tumors, very few have investigated the associations between variants of these genes and the risk of nasopharyngeal carcinoma.

Hildesheim et al genotyped 378 NPC cases and 320 controls employing PCR-RFLP assay and reported that *CYP2E1 *may be a susceptibility gene for NPC development in Taiwan[8]. This study has been cited in the literature over 100 times through the end of 2009. We attempted to replicate this study using the PCR-RFLP assay in our phase I cohort; the bands at agarose gel appeared distinct. However, the analysis results showed that the distribution of genotype was a significant departure from the Hardy-Weinberg equilibrium. Since the TaqMan assay is unavailable for rs2031920, we re-genotyped this SNP by direct sequencing. The results shown in our study are sequencing results (Table 2); however, our findings, which had similar statistical power to the Hildesheim et al study, did not confirm the association observed in the Taiwan study. To further expand our results, an adjacent SNP variant (*CYP2E1*-rs3813867) which tracks the *CYP2E1*- rs2031920 by linkage disequilibrium was investigated in the phase II cohort. The results obtained from this analysis were shown similarly insignificant. From our experience, sequencing assay should be more accurate than the PCR-RFLP. Differences in technique may be the cause of the discrepancies between the results from our study and the Taiwan study[8]. A study conducted in Thailand also showed a slightly increased risk of NPC for *CYP2E1*- rs2031920; again, the result was not statistically significant[9], similar to results of our study.

Over 200 studies have investigated the association between the polymorphism of *GSTP1 *and different cancers, however only one study analyzed this gene region for nasopharyngeal carcinoma. Our results found no significant association between genetic polymorphism of *GSTP1 *and the risk of NPC, similar to a previous report[25]. Our results also showed that there were no significant associations between polymorphisms of *MPO*, *NQO1 *and nasopharyngeal carcinoma in two cohorts (Tables 2, 3).

Our findings are subject to certain limitations. The etiology of nasopharyngeal carcinoma is influenced by many factors, both genetic and environmental. The individual genes we examined appear to have only a limited effect on NPC. However, although our had similar statistical power as Hildesheim et al, due to different study populations, geographic locations, and other unknown factors, our study may not be sufficiently robust to refute the previous study[8]. Additional larger population studies are necessary to determine a definitive answer. Another limitation is that no environmental exposure data (such as smoking history, salt-preserved fish or alcohol consumption, exposure to domestic cooking fires, etc.) is available for our phase I cohort. The environmental exposure data is available for the phase II cohort, and we did conduct an analysis adjusted for salt fish consumption, as well as exposure to domestic cooking fire and occupational solvents, for the phase II genotype data. Still, no significant association was found (data not shown).

## Conclusions

We could not confirm the previously reported increased risk of NPC among individuals homozygous for TT of *CYP2E1*-RsaI (rs2031920) variant. This may be in part due to our use of a different assay method; however, the method we used is now considered the most accurate available. Our results suggest that polymorphism of *CYP2E1*, *GSTP1*, *MPO *and *NQO1 *genes do not contribute appreciably to overall NPC risk in a Han population from southern China. Further studies examining the interaction of genetic and environmental factors on the incidence of nasopharyngeal carcinoma are needed.

## Abbreviations

NPC: Nasopharyngeal carcinoma; SNPS: single nucleotide polymorphisms; RFLP: Restriction fragment length polymorphism; OR: odds ratios; CI: confidence interval; EBV: Epstein-Barr virus; EBV/IGA/VCA: IgA antibodies to Epstein-Barr virus capsid antigen; GSTS: Glutathione S-transferases; MPO: Myeloperoxidase; ROS: reactive oxygen species; IGA+: EBV/IgA/VCA positive; IGA-: EBV/IgA/VCA negative; EBV/IGA/EA: IgA antibodies to EBV early antigen; HWE: Hardy-Weinberg Equilibrium; MAF: minor allele frequencies.

## Competing interests

The authors declare that they have no competing interests.

## Authors' contributions

XG participated in the design of the study, sample collection, performed the statistical analysis and prepared the draft manuscript. YZ organized the sample collection. HD, JL and YZ contributed the cohort collection. JL participated in performing genotype and sequence. BK helped clinic and genotype data management. SJO participated in the study design and coordination and helped to draft the manuscript. All authors read and approved the final manuscript.
